# Dynamic Dual-Branch Encoder and Deformable Spatial Focusing for Accurate Pavement Crack Segmentation

**DOI:** 10.3390/e28070740

**Published:** 2026-07-01

**Authors:** Ruikang Liu, Zixiao Wang, Cheng Zha, Kaijing Song, Lu Hu

**Affiliations:** 1 School of Information and Software Engineering, East China Jiaotong University, Nanchang 330013, China; 2 JiangXi ShineTech Precision Optical Co., Ltd., Xinyu 336600, Chinalu.hu@shine-optics.com (L.H.)

**Keywords:** pavement crack segmentation, deep learning, dual-branch encoder, dynamic feature fusion

## Abstract

Pavement crack segmentation is crucial for enhancing traffic safety, improving maintenance efficiency, extending road lifespan, and supporting smart city development. Utilising computer vision technology to automate crack detection can significantly reduce time and labour costs, improving both accuracy and efficiency. However, pavement crack images exhibit complex visual features, irregular distributions, and diverse shapes and textures, posing challenges for accurate segmentation. To address these issues, a pavement crack segmentation network (PCSNet) based on a dynamic dual-branch encoder and deformable spatial focusing is proposed. The dual-branch encoder employs pre-trained and self-trained branches to extract general and specific crack features, respectively. Dynamic feature fusion optimises the contribution of each branch, enhancing model generalisation. The deformable spatial focusing module refines crack morphological features, improving the model’s ability to identify and localise cracks of varying shapes. Extensive experiments on the DeepCrack dataset show that PCSNet achieves precision, recall, F1 score, and Mean Intersection over Union of 85.34%, 86.16%, 85.75% and 75.23%, respectively, outperforming all comparative methods, thereby validating its superiority.

## 1. Introduction

As the process of urbanization accelerates, traffic volume continues to increase, resulting in an increasing problem of road surface diseases. Pavement crack is one of the most common road diseases, and its formation is usually due to the comprehensive effect of many factors, such as temperature changes, traffic loads and material aging. If the pavement crack is not repaired in a timely manner at the initial stage, it can lead to further deterioration or even more serious damage, resulting in a complex chain reaction of foundation damage and pavement structural instability. This not only greatly reduces the strength and durability of roads, but also increases the risk of accidents, seriously endangers driving safety and affects the efficiency of road traffic. Regular crack detection can identify and treat potential cracks in a timely manner, effectively preventing their further deterioration, thereby significantly improving structural safety and reducing subsequent maintenance costs, which is important for ensuring the safety of public transportation and reducing social and economic costs.

Traditional crack detection mainly relies on manual inspection, which is susceptible to human factors that bias the detection results and make it difficult to ensure their accuracy and consistency. Manual crack detection has obvious shortcomings in terms of efficiency, especially for large-scale pavement crack detection, which often requires a significant investment of manpower and time. In addition, when dealing with small cracks and certain dangerous or inaccessible areas, artificial crack detection is increasingly limited and often does not provide a comprehensive assessment of crack conditions. These deficiencies make it difficult for traditional artificial crack detection to meet the practical application requirements of modern traffic management. Therefore, the study of automatic crack detection has gradually attracted the attention of many scholars, and various methods have been proposed to improve the accuracy and efficiency of crack detection. Notably, training the system requires a dataset with manually placed masks delineating the cracks.

Traditional image processing techniques [1,2,3] are commonly used in crack segmentation tasks, relying on image features such as edge information, texture patterns, and grayscale distribution. However, traditional crack segmentation methods exhibit certain limitations, such as noise sensitivity and poor adaptability to complex backgrounds. With the advancement of deep learning [4] techniques, segmentation methods based on deep learning have made significant breakthroughs in many fields [5,6,7]. To overcome challenges such as poor continuity and low contrast in pavement cracks, Zou et al. [8] developed an end-to-end trainable deep convolutional neural network that automatically detects cracks by learning and extracting high-level crack features. Fang et al. [9] integrated deep learning models with Bayesian probabilistic analysis to tackle the challenge of weak crack signals in noisy backgrounds, thereby significantly enhancing the robustness of crack detection. Xiang et al. [10] incorporated a transformer module to capture contextual information from crack regions, thereby enhancing the accuracy of crack detection. Given the complexity of the pavement background and the variability of cracks, Qu et al. [11] proposed a crack detection algorithm that leverages attention mechanism and multi-feature fusion to enable robust detection across diverse crack patterns and pavement types.

While the aforementioned methods have made notable advances in crack detection, crack images often exhibit complex visual features, making it challenging for a single feature extraction approach to capture all relevant information. In addition, cracks exhibit irregular shapes and diverse morphologies, which complicate the task of achieving accurate crack segmentation. To overcome these challenges, a pavement crack segmentation network (PCSNet) based on dynamic dual-branch encoder and deformable spatial focusing is proposed. The main contributions of this paper are summarized as follows:

(1) A dual-branch feature encoding network is designed to capture general features via transfer learning while simultaneously extracting task-specific features through self-training. This approach enhances the comprehensiveness of feature extraction, which ultimately improves the generalization capability of the model.
(2) The proposed dynamic feature fusion module adaptively adjusts the weights to optimize the representation of fused features, effectively balancing the contributions of general and task-specific features, which in turn enhances the overall performance of the model.
(3) The designed deformable spatial focusing module dynamically adjusts the sampling positions, enabling the model to effectively capture the intricate morphological features of cracks. This enhances the model’s ability to accurately recognize and localize cracks with varying shapes.
(4) PCSNet delivers superior detection performance on DeepCrack. PCSNet demonstrates enhanced accuracy in crack localization and identification, highlighting its potential and advantages for practical applications.

The rest of this paper is organized as follows. Section 2 presents the related work. Section 3 provides a detailed description of PCSNet. Experiments and results are discussed in Section 4. Section 5 presents the conclusions.

## 2. Related Work

With the development of image processing techniques, many traditional image processing methods have been widely used in the field of crack detection. Traditional segmentation methods include edge detection, threshold segmentation, and region growth, among others. These methods analyze texture features and grayscale variations in the image and then identify the crack contours to achieve separation from the background. As the most commonly used edge detection algorithms, Canny [12] detects crack contours by focusing on regions of the image with significant brightness variations. However, it struggles to adequately capture edge details, especially when dealing with weak edges or regions with minimal grayscale variation, resulting in discontinuity in the detected edges. To address these limitations, Zhao et al. [13] employed the Mallat wavelet transform to strengthen the detection of weak crack edges and integrated a genetic algorithm with Canny, significantly improving the accuracy and effectiveness of pavement crack detection. Akagic et al. [14] introduced an unsupervised approach for crack detection by partitioning the input image into four subimages and then identifying cracks by analyzing the ratio between the Otsu threshold and the histogram peak value for each subimage. Zhang et al. [15] proposed an adaptive threshold segmentation method that takes into account the spatial distribution, intensity, and geometric characteristics of cracks, and developed an innovative region growth approach for crack detection.

Deep learning-based segmentation methods [16] have garnered widespread attention from researchers. Long et al. [17] proposed a fully convolutional network that replaces fully connected layers with convolutional layers, allowing arbitrary-sized input images and generating pixel-level outputs matching the input dimensions. However, the information loss due to upsampling operation can lead to blurred edges in the segmentation results. UNet [18] employed a symmetric encoder–decoder architecture and utilized skip connections to merge features from the encoder and decoder to preserve richer details. While skip connections can effectively integrate high level semantic features with low level detailed features, this direct fusion approach may lead to semantic level mismatches and redundant feature interference. UNet++ [19] introduced dense cross-pathway connections to enhance multi-scale feature aggregation, demonstrating improved segmentation accuracy compared to the standard UNet architecture. However, while this improvement enhances performance, it inevitably brings challenges of rising feature redundancy and increasing computational complexity. Attention UNet [20] incorporated an attention mechanism to enhance the feature focusing capability of the model on critical regions, which leads to improved segmentation accuracy and spatial consistency. Nonetheless, it may exhibit performance degradation when dealing with complex scenes containing low-contrast regions. SegNet [21] employed an encoder–decoder architecture that preserves spatial information through max-pooling indices during the decoder phase, achieving memory efficiency while maintaining competitive segmentation accuracy. However, this approach demonstrates performance limitations in handling complex boundary delineations and fine structural details. DeepLab v1 [22], DeepLab v2 [23], DeepLab v3 [24], and DeepLab v3+ [25] are a series of advanced deep learning models developed by Google. DeepLab v3+ enhanced segmentation performance by integrating depthwise separable convolutions with efficient fusion of contextual information, resulting in more precise and computationally efficient segmentation. Gu et al. [26] proposed the Context Encoder Network (CE-Net), which is designed to capture richer high-level features while preserving spatial information, thereby improving the accuracy of image segmentation. Iqbal et al. [27] introduced a double encoder block to effectively capture both local and global context information related to object segmentation. While the integration of contextual information can enhance semantic coherence, it inevitably introduces noise interference that adversely affects segmentation performance, resulting in both boundary blurring and fragmentation of fine-scale structures.

Most existing techniques for pavement crack detection [28] are mostly build upon the aforementioned methods. Sun et al. [29] introduced a multi-scale attention block within the decoder of DeepLab v3+, designed to generate attention masks. Wang et al. [30] developed a hierarchical transformer encoder to extract multi-scale features of cracks and achieved robust segmentation performance in crack of different scales. However, the window partitioning mechanism of transformer may disrupt the continuity of cracks. Yang et al. [31] introduced a triple attention block to capture spatial, channel, and pixel-level attention information. The multi-granularity feature refinement significantly enhances crack segmentation accuracy, albeit at the cost of increased model complexity and computational cost. Al-Huda et al. [32] proposed an asymmetric dual decoder UNet model that incorporates the dual attention module. This design enables the model to effectively capture the features of both thick and fine cracks, even under varying environmental conditions. Zhang et al. [33] employed an approach that combines deformable convolution, weighted loss function, and efficient multi-scale attention module to detect pavement cracks. The hyperparameter sensitivity of the weighted loss function may exacerbate the overfitting of the model to a specific data distribution. Tsai et al. [34] introduced a novel feature refinement module to optimize feature maps, a feature fusion module to effectively combine features, and an attention mechanism to enhance context capture. While the method exhibits high recall rates, its precision performance is notably limited.

## 3. Proposed Method

### 3.1. Overview of PCSNet

**Figure 1:**
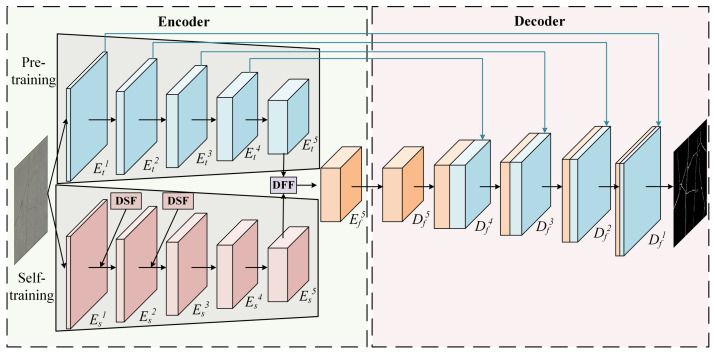
Overall framework of PCSNet.

PCSNet adopts an encoder–decoder architecture, with its detailed structure illustrated in Figure 1. In the feature encoding stage, a pre-trained branch of the encoder is used to capture general features like crack color, edges, and texture, and a self-trained branch integrated with deformable spatial focusing (DSF) module is used to extract detailed features such as crack shape. The dynamic feature fusion (DFF) module is designed to integrate features from the two branches, enabling enhanced coordination and optimization of information across them. In the feature decoding stage, the skip connection mechanism is employed to facilitate the efficient information transfer between the encoder and decoder.

### 3.2. Dynamic Dual-Branch Encoder

**Figure 2:**
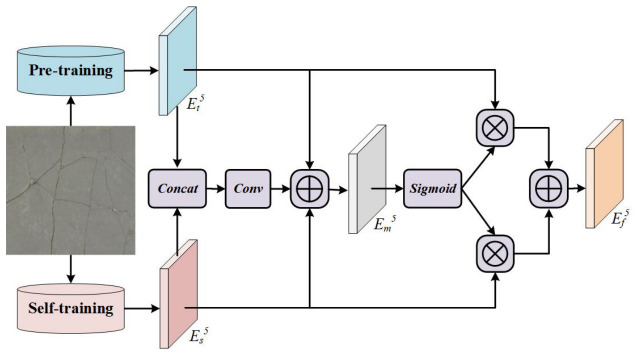
Detailed architecture of DDE.

Crack images typically exhibit a variety of complex visual features. However, single-branch feature extraction networks face challenges in capturing multiple feature types concurrently. To solve this problem, a dynamic dual-branch encoder (DDE) is proposed to capture general features through transfer learning while extracting task-specific features via self-training. To optimize the utilization of features from the dual branches, a dynamic feature fusion (DFF) module is proposed. The detailed architecture of DDE is illustrated in Figure 2.

The high-level features extracted by DDE through the pre-training and self-training branches are denoted by $E_{t}^{5}$ and $E_{s}^{5}$, respectively. Among them, the pre-training branch employs a ResNet50 architecture initialized with parameters pre-trained on the ImageNet dataset, leveraging the generic visual primitives learned from large-scale natural images to provide robust prior guidance for the target domain. This transfer learning approach adopts the pre-trained weights as the initial state of the entire branch, which enables the network to obtain a reasonable parameter starting point at the early training stage and effectively avoids the convergence instability issue caused by random initialization. Meanwhile, it compensates to some extent for the scarcity of labeled samples in the target domain, thereby enhancing the model’s generalization capability and robustness on the downstream task. The high-level features are fused using the concat operation, followed by a convolution operation to adjust the channel dimension. The resulting feature map is then combined with $E_{t}^{5}$ and $E_{s}^{5}$ to generate the intermediate feature representation $E_{m}^{5}$. $E_{m}^{5}$ is passed through a sigmoid function to generate adaptive weight coefficients, which are then applied to $E_{t}^{5}$ and $E_{s}^{5}$ for adaptive fusion, resulting in the fused feature $E_{f}^{5}$. This approach allows the weight coefficients to be dynamically adjusted based on the specific content of the input features, ensuring that the fusion process effectively considers the relative importance of each feature, thereby optimizing the final feature representation. The calculation process of DFF is described by Formulas (1)–(3).

(1) $$E_{m}^{5}=Add\left(Conv\left(Concat\left(E_{t}^{5},E_{s}^{5}\right)\right)\right)$$

(2) $$E_{f}^{5}=\sigma \left(E_{m}^{5}\right)⊗E_{t}^{5}⊕\sigma \left(E_{m}^{5}\right)⊗E_{s}^{5}$$

(3) $$\sigma \left(m\right)=\frac{1}{1+e^{-m}}$$

where *Conv* refers to the convolution operation, σ represents the sigmoid activation function. $E_{f}^{5}$ is obtained as the weighted combination of $E_{t}^{5}$ and $E_{s}^{5}$.

### 3.3. Deformable Spatial Focusing

**Figure 3:**
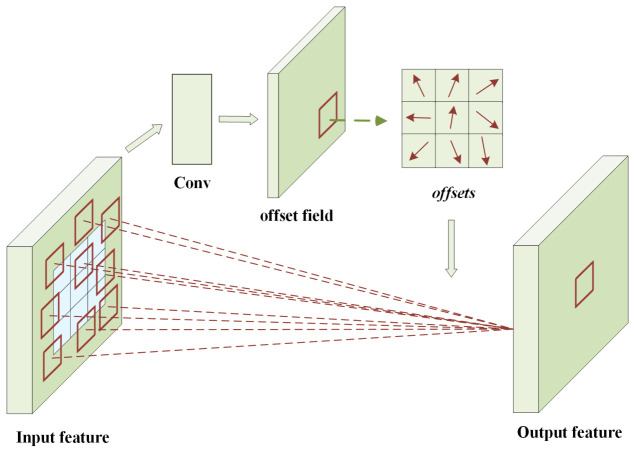
Deformable convolution diagram.

Pavement cracks exhibit complex morphologies and significant irregularities, presenting substantial challenges for the crack segmentation task. To tackle this challenge, a deformable spatial focusing (DSF) module is introduced to effectively handle the diverse and complex shapes of cracks. The module adaptively adjusts the sampling point by incorporating an offset, enabling accurate capture of the subtle features and intricate geometry of the crack. The diagram of the deformable convolution is presented in Figure 3.

The core principle of deformable convolution is to learn spatial offsets within the network, enabling the convolutional kernel to adaptively adjust the sampling positions on the input feature map. These offsets enable the model to selectively focus on specific regions or objects of interest. The deformable convolution modifies the standard sampling by introducing an offset $\Delta P_{n}$, the detailed formulation of which is provided in Formula (4).

(4) $$Y\left(P_{0}\right)=\sum_{P_{n}\in R}W\left(P_{n}\right)*X\left(P_{0}+P_{n}+\Delta P_{n}\right)$$

where $P_{0}$ denotes the center position of the convolution window on the input feature map, $P_{n}$ indicates the relative coordinates of the *n*-th position of the convolution window to $P_{0}$. *R* defines the receptive field size and expansion rate. $W(P_{n})$ represents the weight of the convolution kernel. $X(P_{0}+P_{n})$ refers to the pixel at the *n*-th position within the convolution window.

**Figure 4:**
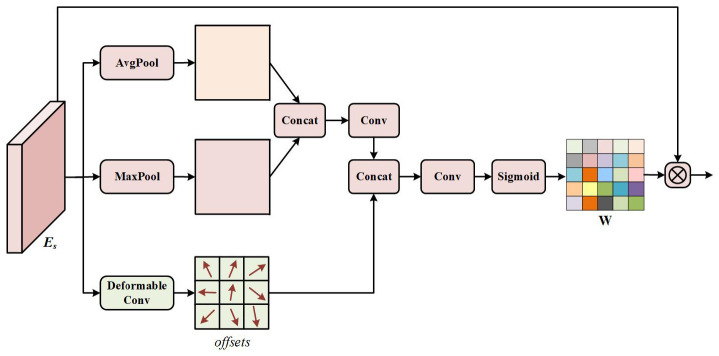
Structure of DSF with the deformable convolution.

To effectively capture the intricate morphological features of pavement cracks, DSF is constructed with the deformable convolution. The detailed architecture of DSF is illustrated in Figure 4. DSF applies max pooling and average pooling along the channel dimension of the input feature map to reduce the channel size, followed by concatenating the resulting pooled features along the channel. A convolution operation is applied to the concatenated results, which are then concatenated with the features extracted via deformable convolution. Subsequently, a convolution operation is applied to the concatenated features and the attention weights are derived through a sigmoid activation function. By assigning varying weights to each spatial position in the input feature map, the representation of the crack features is enhanced, while background regions unrelated to the crack are effectively suppressed. All convolution operations involved in the above process, including those applied to the concatenated features and the deformable convolution, uniformly adopt a kernel size of 3 × 3. The computational procedure of DSF is described by Formulas (5)–(7).

(5) $$D=Conv(Concat\left(AP\left(E_{s}\right),MP\left(E_{s}\right)\right))$$

(6) $$W=\sigma (Conv\left(Concat\left(D,Def\left(E_{s}\right)\right)\right))$$

(7) $$E_{w}=W⊗E_{s}$$

where $E_{s}$ represents the input of DSF. *AP* refers to the average pooling, and *MP* denotes the max pooling. σ represents the sigmoid activation function. *Def* denotes the deformable convolution. $E_{w}$ represents the output of DSF.

### 3.4. Loss Function

The loss function plays an important role in the training process of the network. The core function is to quantify the error between the model output and the true label, and provide a basis for parameter optimization. As the core component of the optimization algorithm, the loss function not only provides the feedback signal to the model, facilitating the network to tune the internal parameters, but also determines the efficiency and convergence of the optimization process. By minimizing the loss function, the model can gradually approach the globally optimal solution, thereby improving the accuracy of crack segmentation.

The Binary Cross Entropy (BCE) loss function effectively distinguishes cracks from the background by optimizing the binary classification result for each pixel. Especially when the background information is dominant, the model can effectively prevent the phenomenon of bias towards the background region. The BCE loss can be expressed by Formula (8).

(8) $$L_{BCE}=-\frac{1}{N}\sum_{i=1}^{N}\left(y_{i}\log\left(p_{i}\right)+\left(1-y_{i}\right)\log\left(1-p_{i}\right)\right)$$

where *N* refers to the total number of pixels, and $y_{i}$ denotes the true label of the *i*-th pixel. $p_{i}$ represents the probability that the model predicts the *i*-th pixel to be a positive sample.

The Dice loss function emphasizes enhancing the overlap between the segmented results and ground truth, thereby leading to more precise and balanced segmentation outcomes. It can be expressed by Formula (9).

(9) $$L_{Dice}=1-\frac{2\sum_{i=1}^{N}p_{i}y_{i}}{\sum_{i=1}^{N}p_{i}+\sum_{i=1}^{N}y_{i}}$$

The integration of the BCE loss function with the Dice loss function can substantially enhance the overall segmentation performance, while maintaining high pixel-level classification accuracy. The total loss function is defined as the combination of BCE loss and Dice loss, which can be mathematically represented by Formula (10).

(10) $$L=L_{BCE}+L_{Dice}$$

## 4. Experiments and Results

### 4.1. Experimental Dataset and Environment

**Figure 5:**
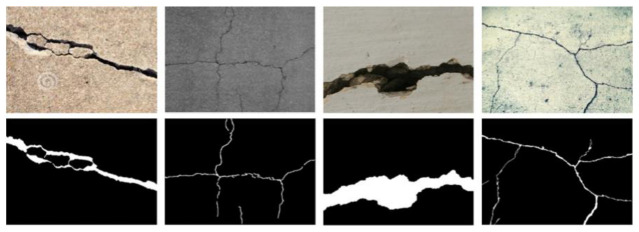
Partial sample diagram of DeepCrack.

DeepCrack [35] serves as a benchmark dataset in the field of crack detection, providing reliable data support for model performance evaluation through its high-quality annotated data and diverse samples featuring low-contrast and complex topological structures. It contains 537 annotated images. A partial sample is presented in Figure 5. The first row displays the crack images, which are JPG images in RGB format with a resolution of 544 × 384. The second row presents the corresponding ground truth mask images. To facilitate effective network training, 377 images are allocated for training and 160 images are reserved for testing. The details of the dataset are given in Table 1. To further substantiate the generalization capability of PCSNet, we conduct experiments on the EdmCrack600 dataset, which encompasses 600 crack images characterized by diverse illumination conditions, pavement categories, and crack morphologies, thereby offering substantial data variability. We compare our method against several state-of-the-art crack detection approaches to thoroughly evaluate its adaptability to varying data distributions.

The proposed method was developed using the PyTorch (2.4.1) framework with key dependencies, including OpenCV, Numpy and Pycocotools. The training and testing processes are conducted on an NVIDIA GeForce RTX 4090D 24 GB GPU, and no special optimization strategy was introduced during the training process. The Adam optimizer is employed during training, with an initial learning rate of 1 $\times \;10^{-3}$ that gradually decays to 1 $\times \;10^{-5}$ over time. The network is trained for 100 epochs with the batch size of 16. The performance of the model is comprehensively evaluated on the test set through multiple evaluation metrics. The experimental configuration is shown in Table 2.

### 4.2. Evaluation Metrics

Upon completion of model training, the segmentation performance of different models on the test set is evaluated using evaluation metrics, including Precision (P), Recall (R), F1 score (F1), and Mean Intersection over Union (MIoU). These metrics can be formally expressed through Formulas (11)–(14).

(11) $$P=\frac{TP}{TP+FP}$$

(12) $$R=\frac{TP}{TP+FN}$$

(13) $$F1=\frac{2^{*}P^{*}R}{P+R}$$

(14) $$MIoU=mean(\frac{TP}{TP+FP+FN})$$

where True Positive (TP) refers to the number of pixels accurately predicted as cracks. False Positive (FP) represents the pixels mistakenly identified as cracks. False Negative (FN) denotes the pixels incorrectly classified as background.

### 4.3. Ablation Experiment

To evaluate the effect of DDE and DSF on enhancing the crack segmentation performance, the ablation experiments are conducted using the controlled variable approach. During the ablation experiments, DDE and DSF are progressively incorporated into the baseline model. To guarantee the fairness and reliability of the ablation experiments, the experiments are performed within a uniform hardware environment. Additionally, the model parameters and training strategies are kept strictly consistent.

The ablation experimental results are presented in Table 3. In this table, ‘w’ indicates that the corresponding model includes the module, while ‘w/o’ indicates that the module is excluded from the model. By comparing the results of the first and second rows, it is evident that the model incorporating DDE demonstrates some improvement across all four metrics, with the R index showing an increase of 2.77%. The observed phenomenon may stem from the pretrained branch extracting generalized features from pavement images via transfer learning, which enhances the model’s sensitivity to faint and blurred cracks while simultaneously increasing false positives in non-crack regions. This led to the phenomenon that R has increased, while P has not improved accordingly. Comparing the experimental results in the second and third rows reveals that the model integrated with DSF achieves further improvement across all four metrics, with the P and MIoU increasing by 2.61% and 2.18%, respectively. This improvement can be attributed to the fact that both DDE and DSF modules enhance the capacity of the model to capture pavement crack features. The ablation experimental results validate the effectiveness of DDE and DSF.

**Figure 6:**
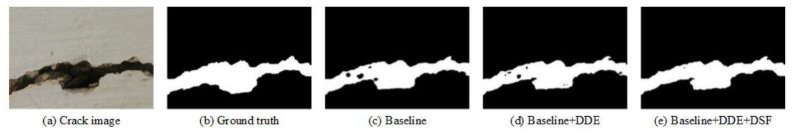
Visual comparions of ablation experiments on DDE and DSF.

**Figure 7:**
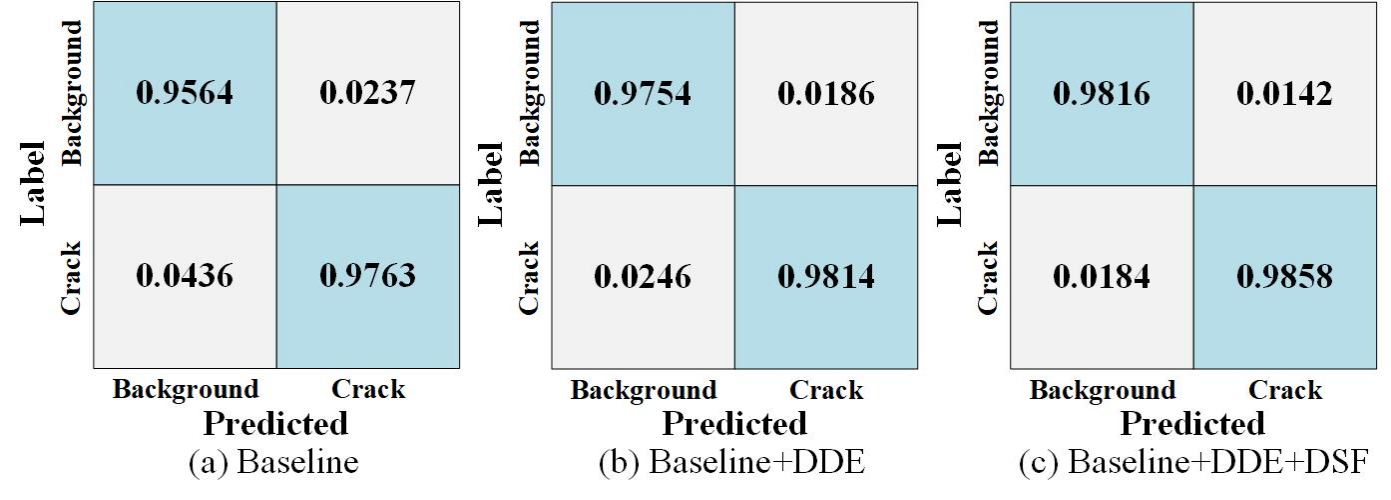
Confusion matrix.

Figure 6 presents the visualization results of the ablation experiment on DDE and DSF. (a) is crack image, and (b) is ground truth. (c) represents the segmentation result of baseline. (d) and (e) are the ablation experiment results on DDE and DSF, respectively. The stone inside the crack has a color and texture similar to the background. As a result, the baseline model mistakenly classifies it as part of the background. In the predicted segmentation mask, this misclassification appears as a black region within the crack. After incorporating DDE and DSF, a notable reduction in the size of the black region inside the crack in the segmentation mask is observed, leading to a significant enhancement in segmentation performance. The segmentation results are quantitatively evaluated using the confusion matrix, as illustrated in Figure 7. As depicted in the figure, the proportion of correctly classified pixels steadily increases with the successive application of DDE and DSF, further confirming the effectiveness of these modules.

### 4.4. Comparison with Other Methods

To demonstrate the effectiveness and superiority of PCSNet, it is compared with several leading image segmentation methods, including CE-Net, MET-Net, DeepCrack, and others. The comparison experiments utilize P, R, F1 and MIoU as evaluation metrics to analyze the performance of these methods on the DeepCrack dataset.

Table 4 presents a comparative performance analysis between PCSNet and other methods on the DeepCrack dataset. The experimental results demonstrate that PCSNet achieves significant advantages across multiple metrics. In terms of P, PCSNet attains 85.34%, outperforming the suboptimal DeepCrack by 2.25%, indicating superior prediction reliability. Regarding R, while BiSeNet achieves the highest value of 92.57%, its P remains at only 78.87%, revealing evident over-segmentation issues. In contrast, PCSNet maintains a balanced performance with 86.16% R while achieving better P-R equilibrium. For comprehensive evaluation metrics, F1 of PCSNet reaches 85.75%, marginally exceeding that of BiSeNet. In terms of MIoU, PCSNet shows leading performance at 75.23%, representing a 1.25% improvement over BiSeNet. Compared with Attention UNet, PCSNet exhibits enhancements of 5.19% in P and 5.74% in MIoU. These quantitative results confirm that PCSNet not only excels in individual metrics but also maintains optimal balance across all metrics, ultimately delivering more stable and reliable crack segmentation performance.

Varying threshold settings can produce different segmentation results, and the robust model is able to adapt to these variations while maintaining consistently high performance. The five thresholds (0.1, 0.3, 0.5, 0.7, 0.9) were strategically selected to cover the full confidence spectrum. Lower thresholds (0.1 and 0.3) prioritize sensitivity for faint crack detection, 0.5 serves as the conventional operating point, while higher thresholds (0.7 and 0.9) test precision optimization by suppressing false positives, thereby comprehensively evaluating the model’s robustness.

Table 5 presents the MIoU of segmentation results for different methods across various threshold values. It is observed that the MIoU for all methods initially rises and then decreases as the threshold is increased. SD denotes the standard deviation of MIoU across different thresholds, with a smaller SD value indicating greater stability of the model. PCSNet exhibits the SD of 0.0415, the lowest among all methods. This can be attributed to its dual-branch architecture, which effectively extracts stable and high-quality features from crack images, enabling the model to maintain superior stability across varying threshold settings.

As shown in Table 6, on the EdmCrack600 dataset, the proposed PCSNet achieves the best performance on both key metrics, with an F1 score of 76.89% and an MIoU of 63.36%, significantly outperforming classic segmentation networks such as UNet, UNet++, and Attention UNet. PCSNet attains a precision of 76.09%, leading all compared methods, which demonstrates its superior capability in suppressing background interference. Meanwhile, it maintains a relatively high recall of 79.85%, achieving a favorable balance between precision and completeness. These experimental results fully validate the effectiveness and generalization capability of the proposed method across different data distributions.

**Figure 8:**
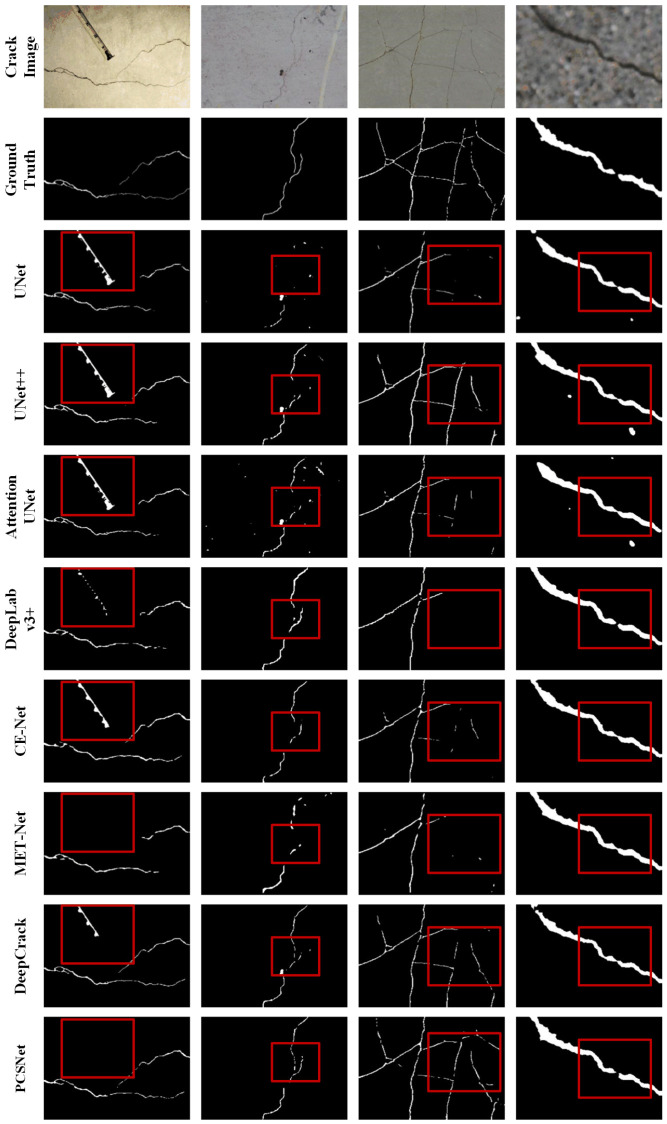
Visualization of pavement crack segmentation results by various methods.

Figure 8 shows the visualization results of pavement crack segmentation using different methods. Upon analyzing the images in the first column, it is observed that all methods except PCSNet and MET-Net incorrectly identify the ruler in the background as a crack. While MET-Net exhibits some resistance to background interference, it encounters issues with missed detections in specific localized regions. In the images shown in the second and third columns, the cracks are relatively small with low contrast against the background, which leads to suboptimal crack segmentation performance for UNet, UNet++, Attention UNet, DeepLab v3+, CE-Net, and MET-Net. These methods struggle to accurately capture the complete crack structure, and some crack edges are not segmented correctly at all. DeepCrack fails to accurately delineate the crack region in the upper right corner of the image in the third column, while PCSNet also exhibits some limitations in segmentation quality within this region. The crack image in the fourth column is blurred. UNet, UNet++, and Attention UNet incorrectly classify background regions as cracks, leading to the appearance of spurious white areas in the segmentation results. While DeepLab v3+, CE-Net, MET-Net, and DeepCrack demonstrate superior performance on blurred images, they still fall short of PCSNet in terms of accurately segmenting crack edges.

To comprehensively evaluate the computational efficiency of each model, we adopts parameter count and floating-point operations (Flops) as evaluation metrics and conducts a comparative analysis of multiple mainstream segmentation methods, with the results presented in Table 7. The experimental results demonstrate that PCSNet achieves significant advantages in both parameter efficiency and computational overhead. Specifically, PCSNet contains 27.27 M parameters, fewer than Attention UNet at 64.24 M, DeepLab v3+ at 39.76 M, and DeepCrack at 30.91 M, while also slightly outperforming CE-Net at 29.00 M and MET-Net at 28.76 M. In terms of Flops, PCSNet requires only 44 GFlops, substantially lower than DeepCrack at 547 GFlops, Attention UNet at 266 GFlops, and DeepLab v3+ at 173 GFlops, with only CE-Net at 36 GFlops being slightly lower. Although CE-Net holds a marginal computational advantage, its segmentation accuracy falls considerably behind that of PCSNet.

## 5. Conclusions

Automated road crack detection technology offers a more efficient and precise solution for road infrastructure maintenance and management. However, Crack images typically exhibit a variety of complex visual features, with pavement cracks exhibiting irregular patterns, diverse shapes, and complex textures, which pose challenges for crack segmentation. To address these issues, a pavement crack segmentation network based on dynamic dual-branch encoder and deformable spatial focusing is proposed. Experiments are carried out on the DeepCrack. PCSNet achieved P of 85.34%, R of 86.16%, F1 of 85.75% and MIoU of 75.23%. PCSNet demonstrated outstanding performance in capturing the complex geometry of cracks and accurately segmenting edge details. It exhibits broad applicability across all stages of modern urban infrastructure development, including construction, inspection and maintenance. By designing a unified data interface and replacing the pre-trained branches with backbones adapted to other domains, PCSNet can be extended to other datasets or projects.

PCSNet enhances the feature extraction ability of the model through a dual-branch network, but it introduces additional parameters and computational overhead, resulting in an increase in the complexity of the model. Future work needs to seek a better balance between model performance and computing efficiency, and explore lightweight technologies such as knowledge distillation, structured pruning, and quantization compression to reduce redundant computing and maintain the competitive advantage of the multi-branch architecture.

## Figures and Tables

**Table 1:** Dateset description of DeepCrack.

Parameter	Value
Crack image format	JPG image in RGB format
Image size	544 × 384
Number of training images	377
Number of test images	160

**Table 2:** Experimental configuration.

Parameter	Value
Batch size	16
Epochs	100
Initial learning rate	1 $\times \;10^{-3}$
Final learning rate	1$\times \;10^{-5}$
Optimizer	Adam

**Table 3:** Effect of DDE and DSF on pavement crack segmentation.

DDE	DSF	P	R	F1	MIoU
w/o	w/o	82.99	83.37	83.18	71.19
w	w/o	82.73	86.14	84.40	73.05
w	w	85.34	86.16	85.75	75.23

**Table 4:** Performance comparison of different segmentation methods on the DeepCrack dataset.

Model	P	R	F1	MIoU
UNet [18]	82.04	82.05	82.04	69.39
UNet++ [19]	80.93	85.48	83.14	70.64
Attention UNet [20]	80.15	83.73	81.90	69.49
DeepLab v3+ [25]	80.04	85.10	82.49	70.32
CE-Net [26]	82.99	83.37	83.18	71.19
MET-Net [27]	80.61	85.91	83.18	71.48
DeepCrack [35]	83.09	83.98	83.53	72.41
BiSeNet [34]	78.87	92.57	85.17	73.98
PCSNet (Ours)	85.34	86.16	85.75	75.23

**Table 5:** MIoU of Segmentation results for different methods at different thresholds on the DeepCrack dataset.

Model	0.1	0.3	0.5	0.7	0.9	SD
UNet	69.38	69.48	69.39	69.20	68.62	0.3106
UNet++	70.22	70.51	70.64	70.69	70.63	0.1696
Attention UNet	68.78	69.29	69.49	69.58	69.39	0.2803
DeepLab v3+	69.74	70.23	70.32	70.19	69.54	0.3068
CE-Net	71.18	71.22	71.19	71.15	71.06	0.0548
MET-Net	70.46	71.19	71.48	71.64	71.39	0.4125
DeepCrack	72.43	72.48	72.41	72.22	71.84	0.2352
BiSeNet	73.57	73.83	73.98	74.11	73.42	0.2819
PCSNet (Ours)	75.19	75.26	75.23	75.23	75.14	0.0415

**Table 6:** Performance comparison of different segmentation methods on the EdmCrack600 dataset.

Model	P	R	F1	MIoU
UNet	59.56	76.39	64.22	49.67
UNet++	59.38	78.32	64.97	50.17
Attention UNet	59.45	79.59	65.71	50.71
DeepLab v3+	64.22	78.77	69.03	54.05
CE-Net	68.54	79.68	72.72	59.00
MET-Net	65.07	80.09	70.82	55.72
DeepCrack	74.31	80.79	76.34	62.67
BiSeNet	73.47	81.99	76.47	62.71
PCSNet (Ours)	76.09	79.85	76.89	63.36

**Table 7:** Comparison of computational complexity.

Method	Params (M)	GFlops
UNet	7.77	55
UNet++	9.16	138
Attention UNet	64.24	266
DeepLab v3+	39.76	173
CE-Net	29.00	36
MET-Net	28.76	132
DeepCrack	30.91	547
PCSNet (Ours)	27.27	44

## Data Availability

Data will be made available on request.
